# Downregulation of PIEZO1 Activity Promotes Breast Cancer Cell Survival under Shear Stress by Modulating β-Catenin and BCL2

**DOI:** 10.1158/2767-9764.CRC-25-0749

**Published:** 2026-07-03

**Authors:** Sreeja Dattachoudhury, Amit Sharma, Renu Sharma, Soumik Pal, Piruthivi Sukumar, Bithiah Grace Jaganathan

**Affiliations:** 1 Stem Cell and Cancer Biology Group, Department of Biosciences and Bioengineering, Indian Institute of Technology Guwahati, Guwahati, India.; 2 Leeds Institute of Cardiovascular and Metabolic Medicine, School of Medicine, University of Leeds, Leeds, United Kingdom.; 3 Jyoti and Bhupat Mehta School of Health Sciences and Technology, Indian Institute of Technology Guwahati, Guwahati, India.

## Abstract

Tumor cells encounter diverse mechanical forces during cancer progression, and the mechanosensitive ion channel PIEZO1 governs how breast cancer cells adapt to these cues, thereby shaping their metastatic potential. Analysis of clinical datasets revealed differential regulation of *PIEZO1* between primary and metastatic breast cancers. In this study, we demonstrate that PIEZO1 regulates the proliferation, migration, and survival of breast cancer cells in a mechanosensitive environment-dependent manner. Under static conditions, optimal PIEZO1 activity is required for both proliferation and migration, which is mediated by metalloproteinases, *RHOA*, and *S100A4*. Although optimal PIEZO1 activity is required for sustained proliferation and migratory capacity, loss of PIEZO1 confers a survival advantage in triple-negative breast cancer cells under mechanically challenging conditions similar to those encountered in circulation. This phenotype was largely associated with context-specific modulation of survival pathways, including β-catenin, pERK1/2, and BCL2, indicating a mechanoenvironment-mediated rewiring of survival pathways. Chemosensitivity assays further revealed that although PIEZO1 silencing sensitized the breast cancer cells to doxorubicin in static conditions, it enhanced drug resistance in anoikis-inducing conditions. Together, these findings uncover a dual, stage-specific role for PIEZO1 in breast cancer, in which optimal PIEZO1 activity sustains proliferation and invasion, whereas PIEZO1 loss enhances cell survival under mechanostress, thereby potentiating metastatic dissemination. These results highlight PIEZO1 as a mechanosensitive protein in breast cancer, and therapeutic modulation of PIEZO1 should consider the breast cancer subtype and mechanical microenvironment for optimal therapeutic outcomes.

**Significance:**

Breast cancer cells encounter diverse mechanical forces during tumor progression and metastasis. We show that although optimal PIEZO1 activity supports growth and invasion at the primary site, its loss enhances survival and drug resistance under shear stress and anchorage-independent conditions, suggesting PIEZO1 as a stage-specific mechanotherapeutic target in breast cancer.

## Introduction

Tumorigenesis is a multistep process that initiates with increased proliferation (hyperplasia/dysplasia) in a confined space, followed by invasion into the surrounding tissue, resulting in distant metastases after entering the circulation. Tumor cells adapt to the changing microenvironments and physical conditions at each step of tumorigenesis. The mechanosensitive ion channel PIEZO1 has emerged as a prognostic marker and potential therapeutic target for several tumor types and is reported to have different roles at different stages of tumor progression (1). Several reports have presented conflicting results about the dysregulation of PIEZO1 expression in breast cancer cells (1–3). Although overall survival and disease-free survival were found to be unaffected by PIEZO1 expression across all breast cancer types, high PIEZO1 levels correlated with worse outcomes, specifically in hormone receptor (HR)–negative breast cancer [estrogen receptor/progesterone receptor (ER/PR)–; ref. 4]. Other studies have also associated high PIEZO1 expression with unfavorable prognosis in patients with breast cancer (3, 5).

Epithelial–mesenchymal transition (EMT) is an important event during the dissemination of tumor cells to distant sites during metastasis (6). Gene set enrichment analysis identified EMT and hypoxia-related pathways as being majorly dysregulated in *PIEZO1*^high^ HR-negative breast cancer (4). Several studies have shed light on the PIEZO1-mediated effects on migration and invasion of breast cancer lines, albeit with contradictory results (2, 5, 7). Compression-induced PIEZO1 activation was shown to reduce 2D migration but increase the invasion capacity of triple-negative MDA-MB-231 and 4T1 breast cancer cells (7). Mechanical stimulation has also been implicated in controlling the growth rate of breast cancer cells. Breast cancer cells have a preference for metastasizing to the bone, which accounts for high morbidity and mortality (8). Mechanical loading of bone has been shown to inhibit bone degradation and tumor burden in SCID mice injected with MDA-MB-231 (9). The role of PIEZO1 in mediating the proliferation of breast cancer cells in response to mechanical cues remains unclear. Intermittent mechanical loading was shown to reduce the proliferation of MDA-MB-231 cells, but not in MCF10A cells with low PIEZO1 expression (10). PIEZO1 activation has been shown to reduce the proliferation and migration of MDA-MB-231 and induce apoptosis (11); however, other studies have reported a contradictory role in regulating the proliferation of breast cancer cells (2, 7).

Mechanistically, PIEZO1 has been suggested to contribute to a hypoxic, glycolytic tumor microenvironment and promote aggressive features through pathways involving WNT/β-catenin signaling in HR-negative breast tumors (4). Low PIEZO1 activity induces the expression of EMT-related genes in lung carcinoma cell lines by increasing Wnt/β-catenin activation (12). ERK phosphorylation is a prominent feature of mechanically stimulated cells; however, its role in PIEZO1-mediated effects is uncertain, as knockdown using siRNA abrogated compression-induced Src2 phosphorylation but further enhanced pERK/ERK levels in breast cancer cells (7).

The advent of the PIEZO1 chemical agonist Yoda1 has enabled modulation of PIEZO1 activity without external mechanical stimulation. Yoda1 is a small molecule that, upon binding to PIEZO1, reduces the threshold of mechanical stimulation required to open the channel, allowing calcium influx (13). Several studies have utilized Yoda1 to probe PIEZO1 function and study the downstream effects across diverse biological contexts, ranging from different cancers to disease models of bone defects and hereditary xerocytosis (14, 15). In cancer models, Yoda1-mediated PIEZO1 activation has been shown to regulate cell proliferation, migration, survival, and mechanotransduction-dependent signaling pathways (11, 12, 16, 17). Yoda1 remains a powerful experimental tool and has been used in this study for dissecting PIEZO1-dependent signaling pathways and mechanotransductive responses in breast cancer cells.

In our study, we elucidated the molecular mechanism by which PIEZO1 mediates migration, proliferation, self-renewal, and survival of breast cancer cells. We hypothesized that PIEZO1 might exert differential effects depending on the subtype and “mechanoenvironment” of the cells. We utilized the 3D-spheroid culture and exposed the breast cancer cells to shear stress (SS) similar to the forces exerted by blood flow during metastasis to model different aspects of tumor progression. In this study, we report a novel role of PIEZO1 as a modulator of self-renewal in breast cancer cells and elucidate the role of the MAPK and β-catenin pathways in mediating the effects of PIEZO1 activation.

## Materials and Methods

### Reagents and cell lines

The primary antibodies against AMPK (cat. #MA515815, RRID: AB_11152993), β-catenin (cat. #138400, RRID: AB_2533039), BCL2 (cat. #MA511757, RRID: AB_10978135), phospho-ERK1/2 (cat. #700012, RRID: AB_2532267), RHOA (cat. #MA1134, RRID: AB_2536840), GAPDH (cat. #398600, RRID: AB_2533438), CD24 (cat. #17-0247-42, RRID: AB_10718833), and EPCAM (cat. #17-5791-80, RRID: AB_2734965), as well as horseradish peroxidase (HRP)–conjugated anti-mouse and anti-rabbit secondary antibodies, were purchased from Invitrogen (Thermo Fisher Scientific). Yoda1 (cat. #SML1558, RRID: SCR_015438) was purchased from Sigma-Aldrich, and BVD-523 (cat. #S7854, RRID: AB_11178658) was purchased from Selleck Chemicals LLC. Fluorescent-conjugated antibodies against phospho-ERK1/2 (cat. #561991, RRID: AB_10895978), phospho-NF-κB (cat. #558423, RRID: AB_647222), phospho-STAT3 (cat. #612569, RRID: AB_399860), phospho-p38MAPK (cat. #612565, RRID: AB_399856), CD44 (cat. #555478, RRID: AB_395870), CD49B (cat. #555498, RRID: AB_395888), and CD49E (cat. #555617, RRID: AB_395984) were purchased from BD Biosciences.

Short tandem repeat profile authenticated and *Mycoplasma*-tested breast cancer cell lines MCF7 (RRID: CVCL_0031; derived from a 69-year-old White/Caucasian female with invasive ductal carcinoma) and MDA-MB-231 [RRID: CVCL_0062; derived from a 51-year-old White/Caucasian female with triple-negative breast cancer (TNBC)] were acquired from the National Centre for Cell Sciences. Upon receipt, the cells were expanded and cryopreserved as master stocks. They were then cultured in Dulbecco’s Modified Eagle Medium (DMEM; Invitrogen, cat. #12100046) supplemented with 10% fetal bovine serum (FBS; Gibco, cat. #A5256701) and 1× penicillin–streptomycin (Invitrogen, cat. #15140122) under standard conditions (37°C, 5% CO_2_). The cells were routinely monitored for morphology and growth characteristics and screened every 2 months for *Mycoplasma* contamination using Hoechst 33258 staining to detect extranuclear DNA. The cells were passaged every 3 to 4 days and used within 10 to 15 passages for all the experiments, after which fresh aliquots from early-passage frozen stocks were thawed to maintain consistency and minimize phenotypic drift.

### Lentiviral transduction

The lentiviral particles were generated by transfecting HEK293FT (RRID: CVCL_6911) cells with pLKO.1 constructs (RRID: Addgene_139470) and packaging plasmids (psPAX2; RRID: Addgene_12260 and pMD2.G; RRID: Addgene_12259) using polyethyleneimine, as described previously (18). Viral supernatants collected at 48 and 72 hours were used to transduce MCF7 and MDA-MB-231 cells in polybrene (4 μg/mL). The scramble and short hairpin RNA (shRNA) against PIEZO1 (shPZ1) silencing plasmids used in the study are from previous reports (19). Stable control and shPZ1 cell lines were obtained via puromycin selection (2 μg/mL).

### Spheroid culture

Breast cancer cell lines MCF7 and MDA-MB-231 were grown in DMEM (Invitrogen, cat. #12100046) supplemented with 10% FBS (Gibco, cat. #A5256701) and penicillin–streptomycin (Invitrogen, 15140122). The 3D spheroids were generated as described previously (20). Briefly, 1,000 cells were seeded in each well of an agar-coated U-bottom 96-well plate (Thermo Fisher Scientific). Yoda1 (10 μmol/L) was added at the time of seeding, and the growth of the spheroid was monitored microscopically using an Axiovert inverted microscope (ZEISS).

### Anoikis and SS assay

Breast cancer cells were cultured in suspension conditions to induce anoikis as described previously (21). A total of 500 cells were seeded in agar-coated 12-well plates to inhibit cell adhesion for 24 hours and then transferred to a six-well plate for colony formation.

To evaluate the effect of SS, the cells were subjected to hydrodynamic SS using an orbital shaker incubator (Scigenics Biotech, Orbitek). SS (τ, in dyne/cm^2^) was calculated using the following equation (22, 23):

$$\tau \;=a\sqrt{{\left(2\pi f\right)}^{3}\eta \rho }$$

where *a* is the orbital radius of the shaker (cm), *ρ* is the density of the culture medium (g/cm^3^), *η* is the viscosity of the medium (Poise), and *f* is the frequency of rotation (rotations/second).

The density and viscosity of DMEM supplemented with 10% FBS were 1.012 g/cm^3^ and 1 mPa·s (10^−2^ Poise), respectively (24), and the orbital amplitude was 25 mm, corresponding to an orbital radius of 1.25 cm. Given that the arterial SS ranges from 5 to 30 dynes/cm^2^ and the venous SS from 0.5 to 4 dynes/cm^2^ (25), and considering that PIEZO1 activation occurs at SS levels exceeding 2 dynes/cm^2^ (26), breast cancer cells were incubated at 100 rpm (1.67 rotations/second), corresponding to an SS of 4.3 dynes/cm^2^, for 24 hours.

Following SS exposure, 2,000 cells per well were seeded for colony formation to assess their self-renewal capacity. The colonies containing more than 50 cells were counted after crystal violet staining using an inverted microscope (ZEISS Axiovert).

### Cell viability assay

Cell viability under anoikis and SS was assessed using annexin V/propidium iodide (PI) staining and flow cytometry. Control adherent (AD), suspension cells, and cells subjected to SS were cultured for 24 hours, stained with annexin V and PI, and analyzed on a BD LSRFortessa. Viable populations (annexin V/PI negative) were quantified using FlowJo (RRID: SCR_008520, FlowJo, LLC).

### F-actin staining

F-actin was stained in control and shPZ1-MCF7 and MDA-MB-231 cells as described previously (21). The cells were cultured on coverslips coated with fibronectin, fixed with formaldehyde (4%), and permeabilized with Triton X-100 (0.5%). F-actin was stained with tetramethylrhodamine isothiocyanate–conjugated phalloidin (Sigma), the nucleus was stained with DAPI, and the cells were imaged using a 20× objective on an Axio Observer inverted fluorescence microscope (ZEISS).

The filopodia density was calculated using the SNT module (22) in ImageJ. The filopodial extension was marked using the path tool. The number of filopodia detected was normalized to the cellular perimeter determined using the freehand line tool to obtain the filopodia density. The mean ± SD filopodia density from *n* ≥ 3 images is presented.

### Ca^2+^ flux assay with Fura2-AM

The intracellular calcium levels were detected using the Fura-2-AM assay. The cells grown to confluency were rinsed with a Ca^2+^-free standard bath solution (SBS; 135 mmol/L NaCl, 5 mmol/L KCl, 1.2 mmol/L MgCl_2_, 1.44 mg/mL glucose, 1.38 mmol/L mannitol, and 0.01 mol/L HEPES). The cells were then loaded with Fura-2-AM (4 μmol/L) in the presence of Pluronic F-127 (1 μg/mL) as a permeabilizing surfactant for 1 hour at room temperature to minimize sequestration of Fura-2 in organelles. Fluorescence emission was recorded at 510 nm in multiple cycles following sequential excitation at 380 nm (F380) and 340 nm (F340), using the shortest possible cycle interval on a Tecan Infinite M200 PRO microplate reader. The F340/F380 ratio, indicative of Ca^2+^-bound Fura-2, was normalized for each well by subtracting the average baseline value obtained over the initial 20 cycles. After baseline recording, the drug-containing SBS with calcium was gently added to the wells using a pipette, reaching a final concentration of 1.5 mmol/L of CaCl_2_ with DMSO alone or with Yoda1, and fluorescence was monitored for an additional 180 cycles. The readings were acquired from three independent wells per condition. The mean and SD values are shown.

### Intracellular calcium measurement using chlortetracycline

The cells (1 × 10^5^ per well) were seeded and cultured under either AD or SS conditions for 24 hours. Chlortetracycline (CTC, 100 μmol/L) was added to the cells for 12 hours prior to the end of incubation. The cells were collected, washed with PBS, and resuspended in growth medium. Equal volumes of cell suspensions were plated into a 96-well plate at 100 μL per well (four wells per condition). Fluorescence was measured using a microplate reader pre-equilibrated to 37°C, with the excitation and emission wavelengths set at 390 and 520 nm, respectively. The readings were obtained from unstained cells and CTC-stained cells to normalize the background. The CTC fluorescence was normalized to the respective AD conditions to control for cell type–specific differences due to dye loading, membrane association, and basal cell state to assess SS-induced changes in calcium homeostasis.

### Proliferation assay

The cells were seeded at equal densities and treated with the indicated concentrations of Yoda1 (0–20 μmol/L). After 72 hours of treatment, they were harvested and stained with 0.4% trypan blue solution. Viable cells (trypan blue–negative) were counted using a hemocytometer to assess proliferation. The cell numbers were normalized to the corresponding untreated control condition. Experiments were performed across at least three independent passages, and representative data are presented as mean ± SE.

### Colony assay

Colony formation assay was performed as described previously (21). During colony formation, the cells were treated with vehicle (DMSO), Yoda1 (10 μmol/L), BVD-523 (5 μmol/L), or doxorubicin (Dox; 1 μmol/L) and allowed to grow. The colonies were fixed with ice-cold methanol (100%), stained with crystal violet, and imaged using an inverted microscope (5× objective, ZEISS). The colonies with more than 50 cells were enumerated, and the colony area was calculated using the freehand selection tool in ImageJ software (RRID: SCR_003070, NIH; ref. 27).

### Migration and spheroid invasion assay

The migration rate of MDA-MB-231 was evaluated via a wound healing assay as described previously (20). The cells were seeded at a density of 20,000 cells/cm^2^ and, after reaching confluency, were serum-starved for 16 hours. A scratch was made, and the cells were treated with DMSO or Yoda1 (10 μmol/L) in 2% FBS. Migration was monitored microscopically, and wound closure at different time points was quantified using T-scratch software, with the migrated area calculated relative to the initial scratch as follows:

$$Migrated\;area=\frac{Open\;area\;at\;0\;hours-Open\;area\;at\;t\;hours}{Open\;area\;at\;0\;hours}\;\times 100$$

The representative data show the average migrated area or migration speed of the cell front over time for *n* ≥ 3 regions, as described previously (20).

The 3D spheroid invasion assay was performed as described previously (20). MCF7 spheroids were formed in agar-coated U-bottom 96-well plates (0.5−2 × 10^4^ cells/mL) and transferred to collagen-coated plates (50 μg/mL) and treated with Yoda1. Cell invasion was monitored using an inverted microscope (ZEISS), and spheroid areas were measured using the freehand selection tool in ImageJ at different time points for a minimum of three spheroids (*n* ≥ 3) per experiment. The area at each time point (t hours) was normalized to the initial spheroid area (0 hours) to calculate percentage migration, as follows:

$$Spheroid\;migration\;area=\frac{Spheroid\;area\;at\;t\;hours}{Spheroid\;area\;at\;0\;hours}\;\times 100$$

### *In ovo* chorioallantoic membrane assay

The *in ovo* chick chorioallantoic membrane (CAM) assay was used as a xenograft model of breast carcinoma. Embryonic day 9 (EOD9) chicken eggs were obtained from the vendor and incubated at 37°C. Eggs were randomly allocated to experimental groups to minimize allocation bias. Because this was a pilot study, a formal power calculation was not required. The air sac was located, and a puncture was made. The eggs were then placed horizontally, and a small puncture was made at the center. Gentle suction was applied through the air sac opening to allow the CAM to detach from the shell. After the CAM dropped, a window of approximately 2 cm in diameter was created on the horizontal surface of the egg. A sterile O-ring was placed on the CAM, and 1 × 10^6^ cells suspended in a medium containing 1× penicillin–streptomycin were seeded within the O-ring. The window was then sealed using sterile surgical tape, and the eggs were incubated for 7 days. On EOD16, the tumors were harvested and imaged. Investigators were blinded to group identity during image acquisition and quantification.

### Cell-cycle analysis

The cells were harvested and fixed overnight at −20°C in 100% ice-cold methanol. Following fixation, the cells were treated with RNase A (100 μg/mL, Invitrogen) for 15 minutes at 37°C and then stained with PI (50 μg/mL). Flow cytometry analysis was performed using a BD LSRFortessa. Cell-cycle distribution (G_0_–G_1_-, S-, and G_2_–M-phase) was determined within the singlet population using the Cell Cycle tool in FlowJo (RRID: SCR_008520, FlowJo, LLC).

### Immunoblotting

The cells were lysed with RIPA buffer [25 mm Tris-HCl (pH 7.4), 150 mm NaCl, 1 mm EDTA, 1% NP-40, 1% sodium deoxycholate, and 0.1% SDS] containing protease inhibitor and phosphatase inhibitor (Thermo Fisher Scientific). Protein concentration was determined using Precision Red reagent (Cytoskeleton Inc.) according to the manufacturer’s protocol, and equal amounts of protein were separated via SDS-PAGE and transferred onto a nitrocellulose membrane using a semidry electroblotting apparatus (Invitrogen). The membranes were incubated overnight with protein-specific primary antibodies and developed with HRP-conjugated secondary antibodies as per the manufacturer’s recommendation. The band intensity for each protein was quantified using Bio-Rad Image Lab Software (RRID: SCR_014210) and normalized to the respective GAPDH levels. Data from ≥3 independent blots are shown as mean ± SD, and group comparisons were analyzed using the Mann–Whitney U test.

### Phospho-flow cytometry and phenotyping

The expression of phospho-ERK1/2 (RRID: AB_399857), phospho-p38 MAPK (RRID: AB_399856), phospho-STAT3 (RRID: AB_647232), and phospho-NFκB (RRID: AB_647222) was determined flow cytometrically as described earlier (28). Briefly, the cells were trypsinized, fixed with 4% paraformaldehyde, and permeabilized with ice-cold methanol. They were stained with fluorescent dye–conjugated antibodies and analyzed on a BD LSRFortessa.

The surface expression of CD24, CD49B, CD49E, and EPCAM was quantified on viable cells using flow cytometry. The adherent cells were detached via brief trypsinization, whereas SS-exposed cells were subjected to an identical trypsinization duration to ensure complete dissociation of cell aggregates. Following immunostaining, PI was incorporated to discriminate live from dead cells during analysis.

### Gene expression analysis

The total RNA was isolated using an RNA extraction kit (Thermo Fisher Scientific) according to the manufacturer’s instructions. cDNA was synthesized using a High-Capacity cDNA Reverse Transcription Kit (Thermo Fisher Scientific) following the manufacturer’s instructions. Real-time PCR was performed using PowerUp SYBR Green (Applied Biosystems) reagent on the Bio-Rad CFX96 thermocycler. The expression levels of GAPDH were consistent between the control and PIEZO1-silenced conditions, as well as between MCF7 and MDA-MB-231 cells, and were subsequently used as the housekeeping gene. The expression level of genes was normalized to the respective GAPDH level and control cells using the ΔΔCt method.

### Statistical analysis

All the statistical analyses were performed using SPSS (RRID: SCR_002865) software. For comparisons between two groups, the Student *t* test was used when data normality was confirmed using the Shapiro–Wilk test, and the nonparametric Mann–Whitney U test was applied when normality could not be established. The qPCR and Western blot analyses were hypothesis-driven and focused on a predefined set of biologically relevant genes and proteins; therefore, statistical comparisons were performed for each gene independently using unpaired two-tailed Student *t* tests. For comparisons involving more than two groups, one-way ANOVA (for normally distributed data) or the Kruskal–Wallis test (for nonnormally distributed data) was performed, followed by appropriate *post hoc* tests. For longitudinal data, two-way ANOVA with repeated measures followed by the Sidak multiple comparisons test was used. *P* values < 0.05 were considered statistically significant.

### Ethics statement

This study does not involve human participants, human-derived tissues, or live vertebrate animals. The *in ovo* chick CAM assay was performed using fertilized chicken eggs prior to EOD16, a developmental stage that is not classified as a live animal under institutional and national guidelines; therefore, animal ethics committee approval was not required. All the cell lines and CAM procedures were performed in accordance with institutional biosafety and handling regulations.

## Results

### PIEZO1 expression is downregulated during breast cancer metastasis

**Figure 1.**
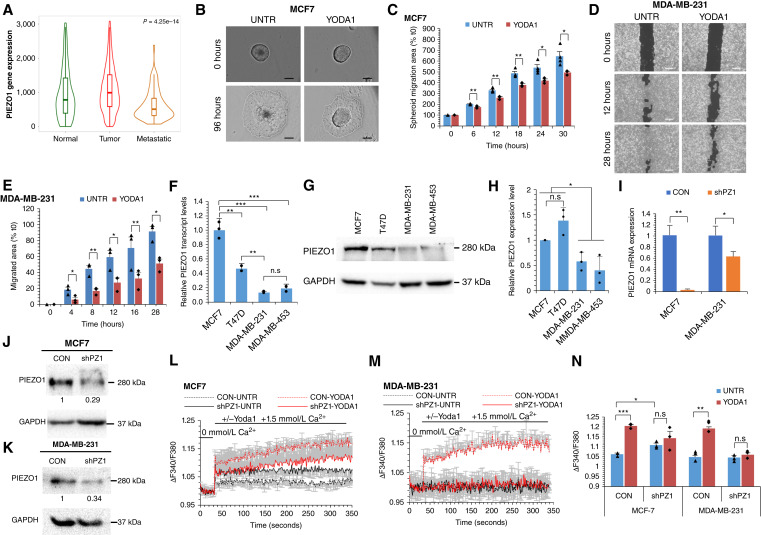
PIEZO1 activation abrogates the invasion and migration capacity of breast cancer cells. **A,** Violin graph showing *PIEZO1* expression levels across normal tissue, primary tumor, and metastatic tumor samples from TNMplot. **B,** Representative micrographs depict the invasion of MCF7 3D spheroids on a collagen matrix. Spheroids were treated with either vehicle (UNTR; *n* = 4) or 10 μmol/L of Yoda1 (YODA1; *n* = 3), with images captured at the indicated time points. The black line in the images represents the scale bar (200 μm). **C,** The migration area of the spheroids shown in **B** is quantified over time and normalized to the initial area at t = 0 hours. Data are presented as mean ± SD with each dot representing an individual spheroid. **D,** Representative micrographs of the wound healing assay at indicated time points showing migration of MDA-MB-231 treated with vehicle (UNTR) or 10 μmol/L of Yoda1 (YODA1). The white line in the images represents the scale bar (200 μm). **E,** Migration speed for MDA-MB-231 described in **D** at different time points. Values are mean ± SD for *n* = 3, with each dot representing a reading from a different well. **F,** The mRNA expression level of *PIEZO1* in a panel of breast cancer cell lines relative to MCF7 cells. Values are mean ± SD for *n* = 3 biological replicates. **G,** Protein expression of PIEZO1 in different breast cancer cell lines. **H,** Densitometric quantification of PIEZO1 normalized to GAPDH in **G**, shown relative to MCF7. Values are mean ± SD for *n* = 3 different blots. **I,** The *PIEZO1* mRNA expression levels in MCF7 and MDA-MB-231 transduced with lentiviral particles containing scramble RNA [control (CON)] or shPZ1. Values are mean ± SD for *n* = 3. **J** and **K,** The expression of the PIEZO1 protein was analyzed in CON and PIEZO1-silenced (shPZ1) MCF7 (**J**) and MDA-MB-231 (**K**). The protein expression levels were normalized to GAPDH levels in the respective conditions. **L** and **M,** The Ca^2+^ flux measured by indicator dye Fura2-AM in CON and PIEZO1-silenced (shPZ1) MCF7 (**L**) and MDA-MB-231 (**M**) treated with vehicle (UNTR) or 10 μmol/L of Yoda1 (YODA1). **N,** The mean peak Ca^2+^ (ΔF340/F380) values obtained in **L** and **M**. Values are mean ± SD for *n* = 3 wells. *P* is shown for two-way repeated measures ANOVA with Sidak multiple comparisons test in **C** and **E**, one-way ANOVA with Tukey multiple comparisons in **F–I**, and two-way ANOVA with Sidak multiple comparisons test in **N**. *, *P* < 0.05; **, *P* < 0.005; ***, *P* < 0.0005.

The expression levels of *PIEZO1* in breast cancer cells at the primary site and metastatic site were first evaluated using TNMplot, which combines publicly available data from The Cancer Genome Atlas and NCBI-Geo (29). We found that *PIEZO1* expression is significantly upregulated in the tumor at the primary site compared with normal tissue (*P* = 0.0000165) but significantly downregulated in metastasized breast cancer cells compared with either normal (*P* = 0.000097) or primary site (*P* < 0.00005) breast cancer cells (Fig. 1A). This downregulation at metastatic sites suggests a possible selective advantage for reduced PIEZO1 activity during dissemination or circulation.

The differential expression of *PIEZO1* prompted us to closely evaluate the role of PIEZO1 in breast cancer under various mechanical environments. Although some studies have explored the role of PIEZO1 in regulating the migration and invasion of breast cancer cells, its molecular mechanism remains unknown.

### Optimal PIEZO1 activity is required for the migration and invasion of breast cancer cells

We modulated PIEZO1 activity in MCF7 (luminal, relatively noninvasive) and MDA-MB-231 (basal/TNBC, highly invasive) cells to test its role in cell motility. Because MCF7 cells have a low migratory capacity in 2D conditions (Supplementary Fig. S1A and S1B), we assessed their invasion potential in a 3D spheroid model within a collagen matrix, as described earlier (21). The invasion ability of MCF7 was significantly reduced when PIEZO1 was activated via Yoda1 treatment (Fig. 1B and C); similarly, the 2D migration ability of MDA-MB-231 was significantly inhibited by PIEZO1 activation (Fig. 1D and E), indicating that sustained PIEZO1 activation can inhibit motility. Supporting this notion, the highly invasive TNBC cell line MDA-MB-231 has significantly lower *PIEZO1* expression compared with the less migratory cell type MCF7 (twofold; *P* < 0.05; Fig. 1F). Similarly, to that seen with MCF7, T47D cells (ER^+^/PR^+^) showed higher *PIEZO1* transcript and protein expression, whereas MDA-MB-453 (ER^−^/PR^−^) cells displayed PIEZO1 levels comparable with those observed in TNBC MDA-MB-231 cells (Fig. 1F–H). Based on these observations, MCF7 and MDA-MB-231 cells, representing the higher and lower ends of the PIEZO1 expression spectrum, respectively, were selected for further investigation.

To determine whether PIEZO1 downregulation enables breast cancer cells to adopt a migratory phenotype, we used lentiviral vectors to silence PIEZO1 expression. Silencing led to a significant reduction in PIEZO1 expression at the transcript and protein levels in both PIEZO1-silenced (shPZ1) MCF7 and MDA-MB-231 cells compared with their respective control vector–transduced cells (Fig. 1I–K). Functional PIEZO1 activity was reduced in both shPZ1 cells, measured by the change in intracellular Ca^2+^ levels upon the addition of the chemical agonist yoda1 (Fig. 1L–N). Furthermore, the mean peak Ca^2+^ signal was higher in unstimulated PIEZO1-silenced MCF7 than in control cells. However, control and shPZ1-MDA-MB-231 showed similar Ca^2+^ levels under unstimulated conditions (Fig. 1N).

**Figure 2.**
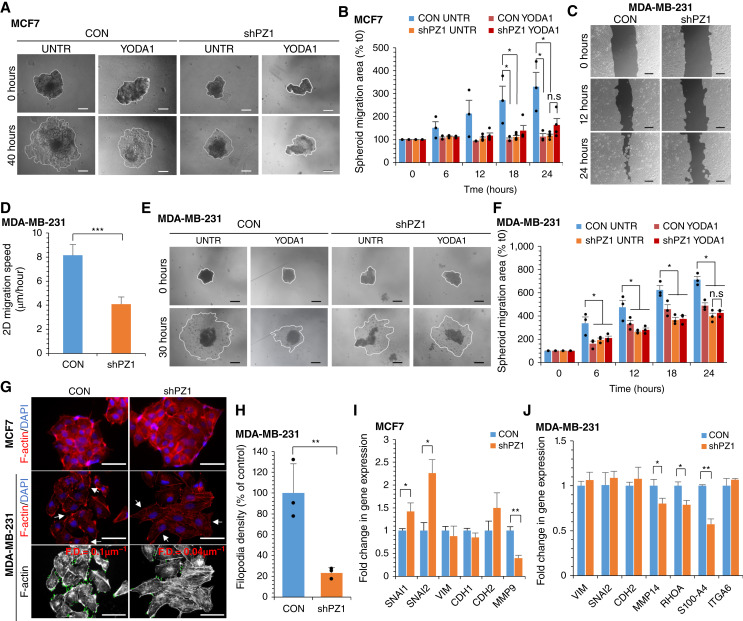
PIEZO1 activity modulates the expression of metalloproteinases and is required for the invasion and migration capacity of breast cancer cells. **A,** Representative micrographs showing the invasion of control (CON; *n* = 3) and PIEZO1-silenced (shPZ1; *n* = 4) MCF7 spheroids treated with vehicle (UNTR) or 10 μmol/L of Yoda1 (YODA1) on a collagen matrix at indicated time points. The white line in the images represents a scale bar (200 μm). **B,** The migration area of spheroids described in **A** at different time points, normalized to the initial area at t = 0 hours. Values are mean ± SD. **C** and **D,** Representative micrographs at indicated time points and a bar graph showing the average migration speed for CON and PIEZO1-silenced (shPZ1) MDA-MB-231 during the wound healing assay. The line in the images represents a scale bar (200 μm). Values are mean ± SD for *n* = 6 different regions. **E** and **F,** Representative micrographs and a bar graph showing the migration of CON and PIEZO1-silenced (shPZ1) MDA-MB-231 spheroids (*n* = 3) treated with vehicle (UNTR) or 10 μmol/L of Yoda1 (YODA1) on a collagen matrix. Values are mean ± SD. **G,** F-actin staining images of CON and PIEZO1-silenced (shPZ1) MCF7 and MDA-MB-231 cells stained for actin (red) with phalloidin–tetramethylrhodamine isothiocyanate and nuclei (blue) with DAPI. White arrows in the middle illustrate the filopodial protrusions in CON and shPZ1 MDA-MB-231 cells. (Bottom) Monochrome images are the F-actin images of CON and shPZ1 MDA-MB-231 cells (middle); the filopodia are depicted by green lines, and filopodia density was calculated using the SNT module of ImageJ (RRID: SCR_003070). The white line in the images represents a scale bar (20 μm). **H,** The filopodia density of MDA-MB-231 described in **G**. Values are mean ± SD for *n* = 4 different regions. **I** and **J,** The gene expression level of EMT and migration-associated genes in CON and PIEZO1-silenced (shPZ1) MCF7 (**I**) and MDA-MB-231 (**J**). Values are mean ± SD for *n* = 3. *P* is shown for two-way repeated measures ANOVA with Sidak multiple comparisons test in **B** and **E**, and unpaired Student *t* test in **D** and **H–J**. *, *P* < 0.05; **, *P* < 0.005; ***, *P* < 0.0005.

Subsequently, we assessed the effect of *PIEZO1* silencing on the invasion capacity of MCF7 and the migration capacity of MDA-MB-231 cells. Interestingly, silencing of *PIEZO1* also reduced the invasion capacity of shPZ1-MCF7 (Fig. 2A and B) and the migration of shPZ1-MDA-MB-231 compared with their respective control cells (Fig. 2C and D). Consistent with the 2D observations, both Yoda1 treatment and *PIEZO1* silencing significantly impaired the invasive capacity of MDA-MB-231 cells under 3D conditions. However, Yoda1 treatment did not further alter the invasive capacity of shPZ1-MDA-MB-231 cells (Fig. 2E and F). To further understand the paradoxical effects of PIEZO1 modulation on breast cancer cell motility, we investigated the changes in morphology, actin arrangement, and expression of migration and EMT-related genes in MCF7 and MDA-MB-231 cells. No remarkable morphologic changes were observed in either shPZ1-MCF7 or shPZ1-MDA-MB-231 cells compared with the control cells. In contrast, pharmacologic activation of PIEZO1 with Yoda1 induced an atypical, elongated morphology specifically in MDA-MB-231 cells, whereas MCF7 cells remained morphologically unaltered (Supplementary Fig. S1C). *PIEZO1* silencing resulted in decreased and disrupted F-actin arrangement in both MCF7 and MDA-MB-231 cells, and there was a decrease in filopodia formation in shPZ1-MDA-MB-231 cells, as evidenced by a lower filopodia density (Fig. 2G and H), consistent with the role of Ca^2+^ influx in cytoskeletal dynamics (30). EMT is a key step in cancer cell invasion and metastatic dissemination, driven by transcription factors such as SNAI1 and SNAI2, which regulate mesenchymal markers including vimentin (VIM) and N-cadherin (CDH2; ref. 6). In agreement with their reduced migration capacity, shPZ1-MDA-MB-231 had a significant reduction in the expression of migration-associated genes *MMP14 *and *S100A4*, but no difference in the expression of *SNAI2*, *VIM*, or *CDH2* was observed. Conversely, shPZ1-MCF7 had significant upregulation of *SNAI1* and *SNAI2* (*P* < 0.05) and a modest increase in *CDH2* levels, accompanied by marked downregulation of the metalloproteinase *MMP9* (*P* < 0.005; Fig. 2I). However, *RHOA*, in which inhibition promoted migration in MDA-MB-231 (31), was significantly downregulated in shPZ1-MDA-MB-231 cells (Fig. 2J). These divergent transcriptional responses reflect the differences in baseline signaling networks between luminal and basal cells, yet converge on the requirement of PIEZO1 for maintaining cell motility. Overall, these results demonstrate that optimal PIEZO1 activity is essential for the intact migration capacity of breast cancer cells, with reduced *PIEZO1* expression impairing the invasion and migration of both luminal-type and basal-like breast cancer cells by modulating the expression of metalloproteinases and invasion-associated genes, such as *S100A4* and *RHOA* GTPase.

### PIEZO1 activity modulates the proliferation of noninvasive breast cancer cells but is dispensable for invasive carcinoma cells

**Figure 3.**
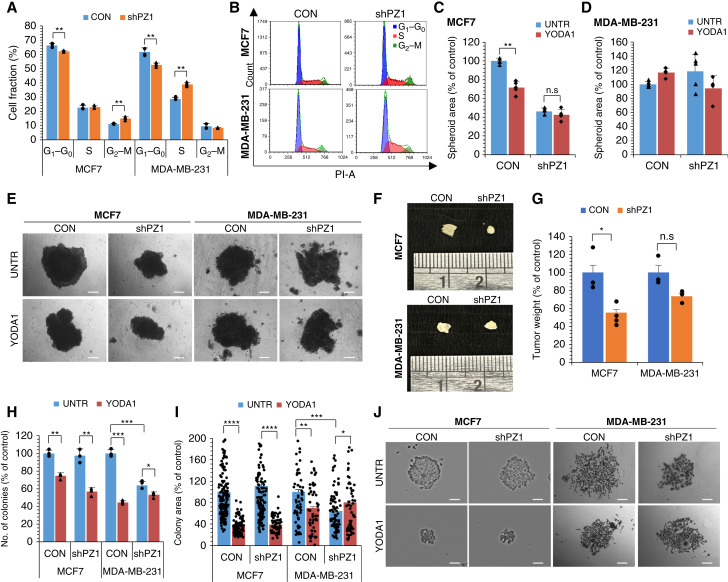
PIEZO1 activity differentially modulates proliferation and self-renewal capacity of MCF7 and MDA-MB-231. **A** and **B,** Bar graph (**A**) and histogram (**B**) for control (CON) and PIEZO1-silenced (shPZ1) MCF7 and MDA-MB-231, showing the distribution of cells in different phases of the cell cycle. Values are mean ± SD for *n* = 3. **C** and **D,** Spheroid growth of CON and PIEZO1-silenced (shPZ1) MCF7 (**C**) and MDA-MB-231 (**D**) treated with vehicle (UNTR) or yoda1 (YODA1) for 14 days. Values are mean ± SD for *n* = 4, with each dot representing an individual spheroid. **E,** Representative micrographs are shown for the spheroid described in **C** and **D**. The line in the images represents the scale bar (200 μm). **F,** Tumor formation was assessed in an *in ovo* CAM assay for CON and PIEZO1-silenced MCF7 and MDA-MB-231. Representative tumor images are shown. **G,** Tumor weights in **F** are shown as a percentage of control for both cell lines. Values are mean ± SD for *n* = 3. **H** and **I,** The number (**H**) and area (**I**) of colonies formed by CON and PIEZO1-silenced (shPZ1) MCF7 and MDA-MB-231 treated with vehicle (UNTR) or Yoda1 (YODA1). Values shown are mean ± SD for *n* = 3 wells in **H** and randomly chosen *n* = 50 colonies in **I**, with each dot representing a different colony. **J,** Representative images of colonies obtained in **H** and **I**. The line in the images represents a scale bar (200 μm). *P* is shown for two-way ANOVA with Sidak multiple comparisons test in **A**, **C**, **D**, **H**, and **I**, and unpaired Student *t* test in **G**. *, *P* < 0.05; **, *P* < 0.005; ***, *P* < 0.0005; ****, *P *< 0.00005.

Next, we examined proliferation and self-renewal as these contribute to tumor growth and recurrence. Under AD conditions, PIEZO1 activation by Yoda1 reduced proliferation in MCF7 and MDA-MB-231 cells in a concentration-dependent manner (Supplementary Fig. S1D). Paradoxically, silencing *PIEZO1* also increased cell-doubling time in both cell lines (55% higher for shPZ1-MCF7 and 29% higher for shPZ1-MDA-MB-231, *P* < 0.005 compared with control cells; Supplementary Fig. S1E). Cell-cycle analysis revealed a modest G_2_–M accumulation in shPZ1-MCF7 and increased S-phase in shPZ1-MDA-MB-231 compared with controls (Fig. 3A and B).

To assess PIEZO1’s role under physiologic conditions, we assessed the 3D spheroid growth after Yoda1 treatment (Fig. 3C–E). Consistent with that observed in 2D conditions, shPZ1-MCF7 formed significantly smaller spheroids than control cells (Fig. 3C and E). Yoda1 treatment reduced spheroid size in control MCF7 but was ineffective in shPZ1-MCF7, indicating that the growth suppression is PIEZO1-dependent. In contrast, modulation of PIEZO1 activity did not affect the growth of MDA-MB-231 spheroids (Fig. 3D and E). To further recapitulate the physiologic conditions associated with *in vivo* tumor growth, we utilized the *in ovo* CAM assay, which has been reported as an effective alternative to immunodeficient mouse models, offering improved stability and reproducibility (32). Similar to that observed in 3D spheroid growth, *PIEZO1* silencing in MCF7 resulted in significantly smaller tumors in the *in ovo* CAM assay (44.7% reduction in tumor weight, *P* < 0.05). In contrast, *PIEZO1* silencing did not significantly affect tumor formation in MDA-MB-231 cells (*P* = 0.1; Fig. 3F and G), suggesting that proliferation and tumor formation are PIEZO1-dependent in MCF7 but not in MDA-MB-231, indicating a subtype-specific effect.

### PIEZO1 activity regulates the self-renewal of breast cancer cells

**Figure 4.**
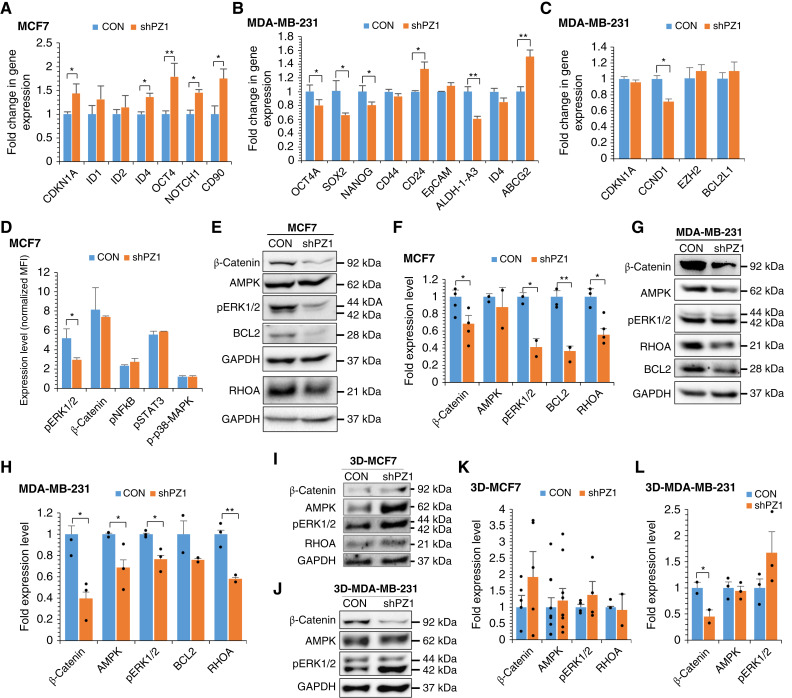
PIEZO1 activity determines the self-renewal capacity of breast cancer cells by modulating the expression of stemness-related genes. **A–C,** The expression levels of stemness-related genes in control (CON) and PIEZO1-silenced (shPZ1) MCF7 (**A**) and MDA-MB-231 (**B** and **C**) were quantified using qPCR. Values are mean ± SD for *n* = 3. **D,** The expression levels of phospho-ERK1/2, β-catenin, NF-κB, phospho-STAT3, and phospho-p38MAPK were determined in CON and shPZ1 MCF7 via phospho-flow cytometry. Values are mean ± SD for *n* = 2. **E–H,** CON and PIEZO1-silenced (shPZ1) MCF7 (**E** and **F**) and MDA-MB-231 (**G** and **H**) were analyzed for the expression of β-catenin, AMPK, pERK1/2, RHOA, and BCL2 via immunoblotting. **I–L,** MCF7 (**I** and **K**) and MDA-MB-231 (**J** and **L**) cells cultured as 3D spheroids were analyzed for the expression of β-catenin, AMPK, phospho-ERK1/2, and RHOA via immunoblotting. Representative immunoblots and densitometric quantifications of *n* = 3–7 blots are shown in **E–L**. Values are mean ± SD. *P* is shown for unpaired Student *t* test in all the panels. *, *P* < 0.05; **, *P* < 0.005.

Breast cancer progression is mediated by self-renewing and therapy-resistant cancer stem cells, which give rise to recurrence and metastasis (33). To assess the role of PIEZO1 in breast cancer self-renewal, we examined the clonogenic potential of MCF7 and MDA-MB-231 cells (Fig. 3H–J). PIEZO1 activation with Yoda1 inhibited colony formation in MCF7 irrespective of the endogenous PIEZO1 expression levels. However, PIEZO1 expression was essential for colony formation in MDA-MB-231, in which *PIEZO1* silencing markedly impaired colony formation. Yoda1 addition impaired the clonogenicity in control cells but not in shPZ1-MDA-MB-231. This indicates that PIEZO1 is dispensable for MCF7 self-renewal but essential for MDA-MB-231 self-renewal. To uncover the molecular basis, we analyzed the genes associated with proliferation and self-renewal in control and shPZ1 cells. In MCF7, *PIEZO1* silencing upregulated the expression of *POU5F1 *(*OCT4*), *NOTCH1*, and *ID1/2/4*, along with increased *CDKN1A* expression (Fig. 4A).

Because CDKN1A enforces cell-cycle arrest and quiescence (34), this transcriptional profile suggests that *PIEZO1* silencing may shift luminal cells toward a quiescent stem-like state, thereby reducing proliferation. However, silencing of *PIEZO1* in MCF7 also resulted in significant upregulation of *CD90*, which is associated with an aggressive basal-like phenotype in breast cancer cells (35). In contrast, MDA-MB-231 cells showed a marked reduction in *OCT4A*, *SOX2*, *NANOG*, and *ALDH1A3* levels with an upregulation of CD24 expression upon *PIEZO1* silencing, indicating a loss of stemness corroborating with the reduced self-renewal observed in shPZ1-MDA-MB-231 (Fig. 4B). Furthermore, *PIEZO1* silencing suppressed *CCND1* expression without altering *CDKN1A* levels (Fig. 4C), indicating the role of PIEZO1 in maintaining the stemness of TNBC cells.

We next examined the signaling pathways associated with proliferation, migration, and survival upon *PIEZO1* silencing. Phospho-flow cytometry analysis revealed no changes in the levels of phospho-NF-κB, phospho-p38MAPK, and phospho-STAT3, but a significant downregulation of phospho-ERK1/2 in shPZ1-MCF7 (Fig. 4D). Immunoblotting revealed reduced levels of β-catenin and BCL2 in addition to reduced pERK1/2 in both shPZ1-MCF7 and MDA-MB-231 (Fig. 4E–H), indicating reduced proliferation under a static mechanoenvironment. However, when cultured as 3D spheroids (Fig. 4I–L), *PIEZO1* silencing reduced β-catenin levels in MDA-MB-231 (Fig. 4J and L), whereas shPZ1-MCF7 retained ERK1/2 and β-catenin levels comparable with controls (Fig. 4I and K).

### SS tolerance is mediated by PIEZO1 downregulation via β-catenin and MAPK signaling

**Figure 5.**
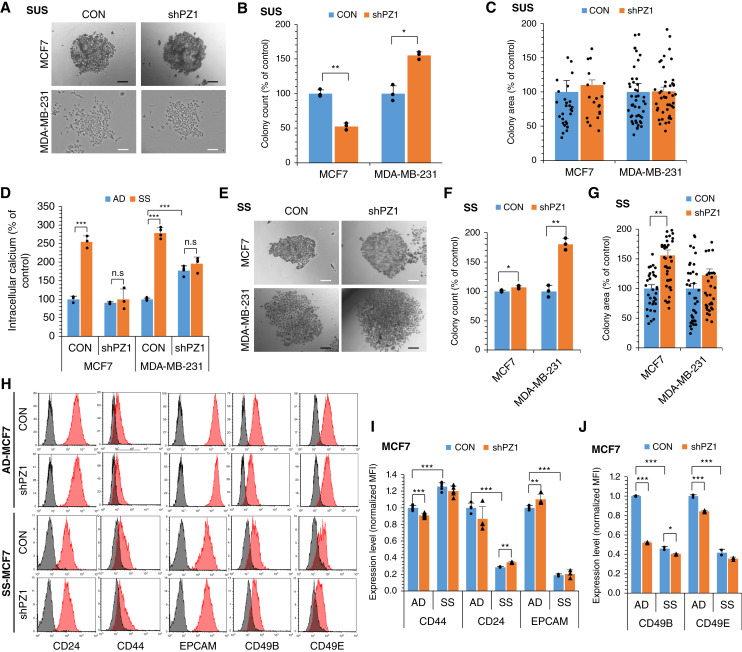
PIEZO1 downregulation promotes survival of SS-exposed breast cancer cells. **A,** Representative images of colonies formed by control (CON) and PIEZO1-silenced (shPZ1) MCF7 and MDA-MB-231 after 24 hours of culture in static suspension (SUS) conditions. The line in the images represents a scale bar (200 μm). **B** and **C,** The colony-forming unit (CFU) number (**B**) and area of colonies (**C**) for MCF7 and MDA-MB-231 are described in **A**. Values are mean ± SD for *n* = 3 in **B** and mean ± SE for *n* = 22–50 colonies in **C**, with each dot representing a different colony. **D,** The total intracellular calcium in CON and PIEZO1-silenced (shPZ1) MCF7 and MDA-MB-231, cultured under static AD or SS conditions, was measured using CTC to assess the effect of PIEZO1 silencing on shear-induced changes in calcium homeostasis. Mean ± SD is shown for *n* = 4. **E,** Representative images of colonies formed by CON and PIEZO1-silenced (shPZ1) MCF7 and MDA-MB-231 after 24 hours of culture in suspension with SS exposure as described in the “Materials and Methods” section. The line in the images represents a scale bar (200 μm). **F** and **G,** The CFU number (**F**) and area of colonies (**G**) for SS-exposed MCF7 and MDA-MB-231 are described in **E**. Values are mean ± SD for *n* = 3 in **F** and mean ± SE for *n* = 40–50 colonies in **G**, with each dot representing a different colony. **H,** The representative histograms show the surface expression of stemness markers and integrins, assessed via flow cytometry, for AD cultured and SS exposed CON and PIEZO1-silenced (shPZ1) MCF7 cells. **I** and **J,** Graphs showing mean fluorescence intensity (MFI) normalized to the respective AD control condition for each marker shown in **H**. The values are mean ± SD. *P* is shown for unpaired Student *t* test in **B**, **C**, **F**, and **G**, and for two-way ANOVA with Sidak multiple comparisons test in **D**, **I**, and **J**. *, *P* < 0.05; **, *P* < 0.005; ***, *P *< 0.0005.

Considering the reduced expression of *PIEZO1* in breast cancer cells at metastatic sites, we hypothesized that PIEZO1 activity might mediate the tolerance of breast cancer cells against anoikis (cell death due to loss of adhesion) and SS experienced during metastasis. Because the ability of cells to form colonies under anchorage-independent conditions is a well-established indicator of metastatic potential (36), breast cancer cells were subjected to anoikis-inducing suspension conditions, and their colony-forming ability was tested. Anoikis induction was evident from the reduced viability of MCF7 cells under suspension conditions (Supplementary Fig. S1F). Although shPZ1-MCF7 cells displayed modestly higher viability than control cells, they formed significantly fewer colonies following anoikis induction, with no difference observed in colony size (Fig. 5A–C). In contrast, shPZ1-MDA-MB-231 cells subjected to anoikis-inducing conditions formed significantly more colonies than control cells, with no difference in their colony area, indicating that downregulation of PIEZO1 increases anchorage independence in the invasive TNBC but not in the luminal cells.

During metastasis, circulating cancer cells must adapt to the SS generated by the blood flow. When subjected to SS conditions mimicking the vascular environment, both MCF7 and MDA-MB-231 cells showed a significant reduction in viability compared with static AD controls (Supplementary Fig. S1F and S1G). To confirm PIEZO1 modulation in the SS model, we evaluated the changes in intracellular Ca^2+^ levels using CTC, which gives fluorescence upon Ca^2+^ binding (37). SS induced a marked increase in Ca^2+^ levels in control MCF7 and MDA-MB-231 cells, whereas this response was blunted in PIEZO1-depleted cells, indicating that PIEZO1 contributes to shear-induced intracellular Ca^2+^ elevation as measured using the endpoint fluorescence assay (Fig. 5D). CTC revealed modestly higher baseline intracellular Ca^2+^ levels in shPZ1-MDA-MB-231 cells, similar to the trend observed in shPZ1-MCF7 cells using Fura-2 (Fig. 1L and N), suggesting that different Ca^2+^ indicators vary in their sensitivity for detecting subtle quantitative differences. Notably, CTC detects the total intracellular Ca^2+^, including membrane-associated and intracellularly sequestered pools, making it suitable for quantifying sustained alterations in calcium homeostasis induced by *PIEZO1* silencing. In contrast, Fura-2 is optimal for the ratiometric detection of transient cytosolic Ca^2+^ elevations.

When subjected to SS, both MCF7 and MDA-MB-231 cells with *PIEZO1* silencing exhibited better preservation of colony-forming ability. Specifically, shPZ1-MDA-MB-231 cells generated significantly more colonies, whereas shPZ1-MCF7 cells produced notably larger colonies (Fig. 5E–G), indicating that *PIEZO1* silencing enhances repopulation capacity under mechanically challenging conditions. To understand the molecular mechanisms underlying increased shear tolerance upon *PIEZO1* silencing, we assessed stemness and migration-related markers in MCF7 cells. Exposure to SS significantly reduced CD24 and EPCAM expression in both control and shPZ1-MCF7 cells (Fig. 5H and I). Because integrins mediate the interaction of cells with the extracellular matrix and transduce physical cues (38), we evaluated integrin expression under these conditions. *PIEZO1* silencing significantly decreased CD49b and CD49e expression under AD conditions (Fig. 5H and J). Upon exposure to SS, control-MCF7 showed a marked reduction in the expression of CD49b and CD49e, whereas shPZ1-MCF7 maintained comparable expression levels to that of AD conditions, indicating that PIEZO1 modulates integrin-mediated responses against shear stimulus. These findings suggest that the downregulation of adhesion molecules, such as CD24, EPCAM, and integrins, represents a survival adaptation to SS, with *PIEZO1* silencing preconditioning cells through reduced integrin expression.

**Figure 6.**
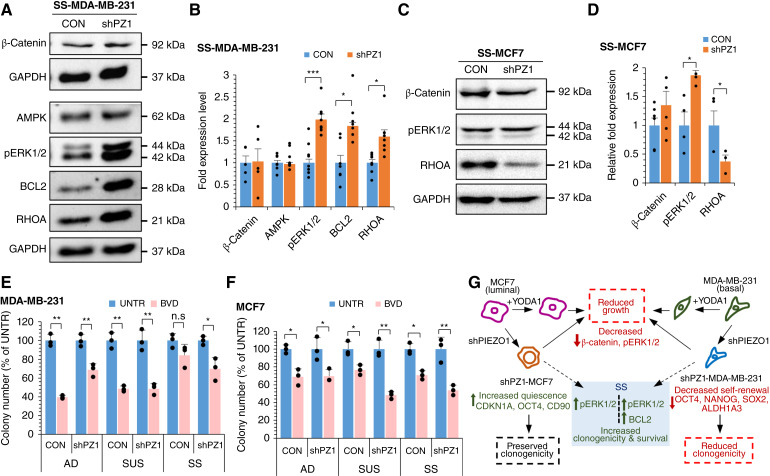
PIEZO1 activity modulates β-catenin and MAPK signaling in breast cancer cells under SS conditions. **A,** Representative data for SS-exposed control (CON) and PIEZO1-silenced (shPZ1) MDA-MB-231 analyzed for the expression levels of β-catenin, AMPK, phospho-ERK1/2, BCL2, and RHOA via immunoblotting. **B,** The densiometric quantification of β-catenin, AMPK, pERK1/2, BCL2, and RHOA normalized to GAPDH levels for immunoblots described in **A**. Values are mean ± SD for *n* = 3–8 different blots. **F** and **G,** Representative immunoblots (**C**) and GAPDH normalized densiometric quantification (**D**) showing expression levels of β-catenin, p-ERK1/2, and RHOA in SS-exposed CON and PIEZO1-silenced (shPZ1) MCF7. Values are mean ± SD for *n* = 3–5 blots. **E** and **F,** Colony numbers obtained for CON and PIEZO1-silenced (shPZ1) MDA-MB-231 (**E**) and MCF7 (**F**) treated with vehicle (UNTR) or 10 μmol/L of BVD-523 (BVD) under AD, static suspension (SUS), or SS conditions. Values are mean ± SD for *n* = 3. **G,** Schematic summarizing the modulation of stemness and proliferation in breast cancer cells upon PIEZO1 modulation. *P* is shown for the Student *t* test in all the panels. *, *P* < 0.05; **, *P* < 0.005; ***, *P* < 0.0005.

Given the increased SS tolerance in shPZ1-MDA-MB-231, we assessed the signaling pathways involved in survival and proliferation. In contrast to basal 2D conditions, under SS, shPZ1-MDA-MB-231 showed elevated pERK1/2, BCL2, and RHOA levels compared with control cells (Fig. 6A and B). In contrast, β-catenin levels were unchanged, and increased pERK1/2 and reduced RHOA were seen in shPZ1-MCF7 cells (Fig. 6C and D). To determine whether the differential effects of *PIEZO1* silencing on clonogenicity under distinct mechanical conditions were mediated by ERK1/2, we treated MCF7 and MDA-MB-231 cells with the ERK1/2-specific inhibitor BVD-523 (BVD). BVD treatment reduced clonogenicity in both control and PIEZO1-silenced MDA-MB-231 and MCF7 cells under AD conditions, although shPZ1-MDA-MB-231 cells showed a comparatively reduced response to BVD treatment (Fig. 6E and F). However, under static suspension conditions, control and shPZ1-MDA-MB-231 showed similar sensitivity to BVD, whereas shPZ1-MCF7 showed higher sensitivity. When subjected to SS, a significant reduction in colony numbers was observed with BVD treatment in both shPZ1-MDA-MB-231 and shPZ1-MCF7 cells, indicating that the elevated pERK1/2 levels observed in shPZ1 cells are critical for preserved clonogenicity under SS. Together, these results indicate that *PIEZO1* silencing enhances SS tolerance in TNBC cells by modulating pERK1/2 and BCL2 levels, thus favoring metastatic survival. These results further highlight the contextual role of PIEZO1: Although its loss broadly impairs β-catenin and MAPK signaling in 2D, its effects are modulated by the mechanoenvironment, differentially shaping self-renewal and migration in luminal versus basal-like breast cancer cells (Fig. 6G).

### PIEZO1 modulates the sensitivity of breast cancer cells to Dox

**Figure 7.**
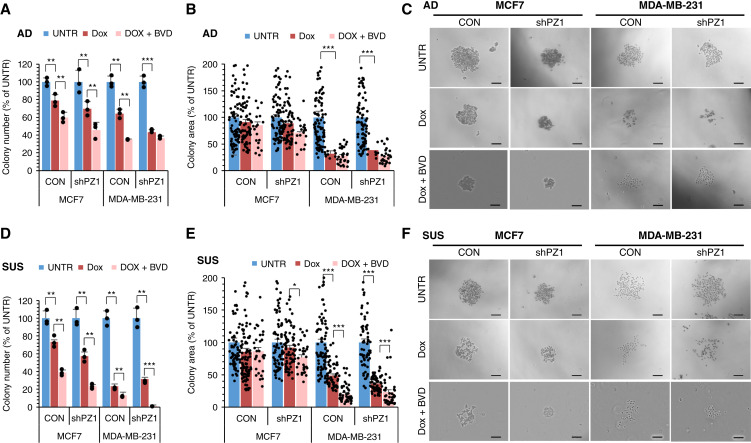
PIEZO1 activity differentially modulates the chemosensitivity of breast cancer cells depending on the molecular subtype and mechanical cues. **A–C,** The colony-forming ability of CON and PIEZO1-silenced (shPZ1) MCF7 and MDA-MB-231 cells was assessed in the absence (UNTR) or presence of Dox with and without BVD-523 (BVD) treatment. The number (**A**) and total area (**B**) of colonies were quantified and normalized to their respective untreated controls to delineate the contribution of PIEZO1 activity to Dox-induced inhibition. **D–F,** The Dox-induced inhibition of clonogenic potential was further evaluated in CON and shPZ1 MCF7 and MDA-MB-231 cells cultured under suspension conditions. Colony number (**D**) and colony area (**E**) are shown as a percentage of the corresponding untreated (UNTR) condition (*n* = 3 wells for **A** and **D**; *n* = 20–30 colonies per condition for **B** and **E**). *P* values are shown for two-way ANOVA with Sidak multiple comparison test in all the panels. *, *P* < 0.05; **, *P* < 0.005; ***, *P* < 0.0005.

Given that *PIEZO1* silencing modulates the proliferation and self-renewal of breast cancer cells, we next investigated how PIEZO1 influences their response to chemotherapy. Dox induces DNA damage–mediated apoptosis and remains a central component of standard chemotherapeutic regimens for breast cancer, particularly in anthracycline-based combinations such as adriamycin–cyclophosphamide and 5-fluorouracil–adriamycin–cyclophosphamide; however, resistance remains a major therapeutic challenge. Furthermore, under certain contexts, Dox treatment itself can lead to drug resistance and an adverse metastatic tumor phenotype (39). PIEZO1 has been implicated in modulating drug response across multiple cancers (40), suggesting that its activity could shape the subtype-specific chemosensitivity of breast cancer cells. *PIEZO1* silencing significantly enhanced the sensitivity of both MCF7 and MDA-MB-231 cells to Dox treatment, resulting in a significant reduction in colony-forming ability. Although Dox significantly inhibited colony formation in both cell types, the inhibitory effect with *PIEZO1* silencing was significantly higher compared with the control conditions in both MCF7 and MDA-MB-231. Notably, BVD treatment enhanced Dox sensitivity in both control- and shPZ1-MCF7 and control MDA-MB-231 but not in shPZ1-MDA-MB-231 (Fig. 7A–C).

When subjected to anoikis-inducing suspension conditions, shPZ1-MCF7 cells had higher Dox sensitivity, with a pronounced reduction in colony-forming ability. In contrast, consistent with their behavior in untreated anoikis conditions (Fig. 3B), *PIEZO1* silencing in MDA-MB-231 promoted survival in the anchorage-independent conditions and reduced chemosensitivity under Dox treatment conditions (Fig. 7D–F). Although Dox treatment inhibited proliferation, resulting in reduced colony area, silencing *PIEZO1* did not lead to further changes in the colony area compared with the control conditions. However, when treated with BVD under anoikis conditions, BVD exerted a stronger inhibitory effect on colony formation in shPZ1-MDA-MB-231 cells. Similarly, under SS conditions, BVD suppressed the survival of both shPZ1-MCF7 and shPZ1-MDA-MB-231 cells in both Dox-treated and untreated conditions, indicating that pERK1/2 signaling mediates prosurvival effects during PIEZO1 silencing (Supplementary Fig. S1H and S1I). These findings indicate that PIEZO1 modulates Dox sensitivity in a subtype- and context-dependent manner, differentially influencing the chemoresponse of luminal (MCF7) and basal-like (MDA-MB-231) breast cancer cells by modulating survival signaling pathways.

## Discussion

The mechanical properties of the tumor microenvironment play an important role in breast cancer, with the mechanosensitive proteins enabling the tumor cells to adapt to various stages of tumor progression during cancer progression and metastasis. In this context, PIEZO1, a mechanosensitive ion channel protein, has emerged as a key mechanosensitive protein in regulating the migration and invasion of breast cancer and other cancer types. Our findings reveal that PIEZO1 regulates the dynamic balance between proliferation, stemness, migration, and metastasis in a context-dependent manner, which explains the conflicting conclusions on the role of PIEZO1 reported by previous studies (2, 10, 11, 41).

We found that *PIEZO1* expression is significantly lower in metastatic MDA-MB-231 cells compared with MCF7, which represent ER^+^PR^+^ breast cancer cells (2). Modulating PIEZO1 activity, either through Yoda1-mediated activation or gene silencing, inhibited proliferation in both MCF7 and MDA-MB-231 cells, suggesting that an optimal range of PIEZO1 activity is required to maintain proliferative homeostasis. However, PIEZO1 showed divergent effects on cellular function; in MCF7, *PIEZO1* silencing upregulated pluripotency and quiescence-associated genes, such as *OCT4*, *NANOG*, *ID1/2/4*, and *CDKN1A*, suggesting a shift toward a nonproliferative quiescent state. In MDA-MB-231, loss of PIEZO1 resulted in downregulation of *OCT4A*, *SOX2*, *NANOG*, and *ALDH1A3*, suggesting diminished self-renewal and clonogenic potential.

PIEZO1 also regulated the migration ability of breast cancer cells through regulation of the cytoskeleton and extracellular matrix remodeling pathways. Loss of migration ability due to *PIEZO1* silencing was associated with downregulation of matrix metalloproteinases, which play a critical role in cell invasion by degrading ECM and enabling invadopodium formation (42). MCF7 cells, as highly differentiated luminal breast cancer cells, exhibit strong epithelial characteristics, including high CDH1 expression and low basal levels of EMT transcription factors such as SNAI1 and SNAI2. Although shPZ1-MCF7 cells showed increased *SNAI1/2* expression, the absence of significant upregulation in downstream EMT markers (e.g., *VIM* and *CDH2*) suggests that SNAI1/2 levels were insufficient to drive full EMT. Additional pro-EMT pathways, such as TGF-β signaling, may be required to potentiate EMT induction (43). Notably, whether PIEZO1 activity influences TGF-β signaling remains an open question. The reduction in migration of MDA-MB-231 was accompanied by a significant decrease in the expression of *S100A4*, a Ca^2+^-binding metastasis-associated protein associated with aggressive disease (44, 45). The decreased migration was accompanied by the suppression of RHOA (46, 47) and downregulation of mesenchymal markers CD44, CD49b, and CD49e in MCF7, which are known regulators of stemness and migratory ability in breast cancer (48–51). The inhibitory effects of Yoda1 on the migration and proliferation of breast cancer cells may also be attributed to the inhibitory effects of Ca^2+^ overload, as observed in epithelial cells (52). Taken together, and consistent with previous studies demonstrating that optimal PIEZO1 activity is required for the maintenance of cellular functions in other cell types (19), our findings indicate that both excessive activation and loss of PIEZO1 impair the migratory capacity of breast cancer cells.

PIEZO1 activity had varied effects on the proliferation and self-renewal of MCF7 and MDA-MB-231 cells. Epithelial homeostasis has been shown to be mechanically regulated by PIEZO1 activation, inducing cellular proliferation or apoptosis depending on the cell crowding (53). Under normal 2D culture conditions, PIEZO1 activation and silencing inhibited proliferation in both MCF7 and MDA-MB-231 cells. In contrast, in 3D spheroid cultures that better mimic *in vivo* tumor growth, altering PIEZO1 activity, either through Yoda1 treatment or silencing, reduced 3D spheroid growth of MCF7 but not MDA-MB-231. This effect was also pronounced in the *in ovo* tumor growth, in which reduced tumor size was observed in PIEZO1-silenced MCF7 and not in MDA-MB-231.

The mechanical context of the microenvironment modifies the role of PIEZO1 in breast cancer cells. Exposure to SS, which the breast cancer cells encounter during metastatic circulation, induced upregulation of CD44 and downregulation of CD24 and EPCAM in MCF7, regardless of PIEZO1 expression levels. However, under similar conditions, silencing of *PIEZO1* significantly decreased RHOA levels in MCF7 but increased RHOA levels in MDA-MB-231, indicating a subtype-specific effect based on the migratory status of the cells. Although PIEZO1-induced variations in RHOA expression levels suggest its involvement in cell migration, determining how PIEZO1 influences the active GTP-bound RHOA levels is required to understand the context-dependent role of PIEZO1.

The contrasting effect of PIEZO1 in different breast cancer subtypes was more pronounced in conditions that mimic metastatic transit. During this transition, the mechanical cues experienced by tumor cells change dramatically as the cells adapt to resist cell death due to loss of adhesion (anoikis) and SS exerted via blood flow (54). In static conditions, *PIEZO1* silencing did not affect the self-renewal capacity of MCF7 but reduced that of MDA-MB-231, which may be attributed to increased expression of *OCT4* in MCF7 but a concomitant reduction in *OCT4*, *NANOG*, *SOX2*, and β-catenin and phospho-ERK1/2 levels in MDA-MB-231. In contrast, under anoikis-inducing conditions, *PIEZO1* silencing sensitized MCF7 to loss of adhesion and reduced their clonogenic ability, whereas shPZ1-MDA-MB-231 cells acquired a survival advantage and showed higher colony-forming ability and increased expression of survival-associated proteins such as β-catenin, pERK1/2, and BCL2. Furthermore, after exposure to SS, MDA-MB-231 cells showed increased colony formation in an ERK1/2-dependent manner, confirming the finding that PIEZO1 downregulation promotes metastatic survival of TNBC cells under mechanically stressful conditions.

Chemosensitivity assays further confirmed the context-dependent role of PIEZO1 in breast cancer. Under static AD conditions, *PIEZO1* silencing sensitized both MCF7 and MDA-MB-231 to Dox treatment and reduced the clonogenic ability more than Dox treatment alone. However, under anoikis-inducing conditions, PIEZO1 downregulation sensitized MCF7 cells to Dox, whereas MDA-MB-231 exhibited an attenuated response. This resembles the survival advantage observed under SS, in which PIEZO1 downregulation rewires the mechanosensitive pathways in MDA-MB-231 cells, resulting in decreased chemosensitivity during metastatic transit. ERK1/2 inhibition could attenuate this survival advantage, particularly in PIEZO1-silenced TNBC MDA-MB-231 cells.

Mechanistically, assays to quantify PIEZO1 activity during dynamic SS conditions are limited. The current study assessed PIEZO1 activity under SS conditions using CTC as an indicator of induced calcium currents. Although Fura-2 AM is ideal for capturing cytosolic calcium spikes, its requirement for a short loading window followed by wash steps introduces additional mechanical handling that could alter the shear-induced state. In contrast, long loading periods are routinely used for CTC, allowing quantification without the processing artifacts or signal loss to efflux and compartmentalization. However, CTC has limited quantitative sensitivity and lower temporal resolution, and it cannot capture real-time PIEZO1 activity and transient flux triggered by the onset of SS. For such dynamic measurements, the use of genetically encoded calcium indicators like GCaMP or electrophysiologic approaches would be required, as they may allow continuous, high-resolution monitoring of cytosolic calcium fluctuations throughout the entire 24-hour mechanical stimulation period without the need for exogenous dye loading or washing (37). Furthermore, our observation that PIEZO1-silenced cells maintain higher basal Ca^2+^ levels than control cells suggests compensatory upregulation of other Ca^2+^ ion channels, as seen in Piezo1-knockout muscle stem cells (55). Such adaptive responses complicate the interpretation of endpoint calcium measurements and underscore the importance of future studies incorporating real-time calcium imaging to fully delineate PIEZO1-specific mechanotransduction under SS. In addition, although this study focuses on two well-characterized breast cancer cell lines of distinct molecular subtypes (luminal MCF7 and basal/triple-negative MDA-MB-231), validation across additional cell lines, patient-derived models, and *in vivo* mammalian systems can establish the generalizability of PIEZO1-dependent phenotypes, particularly with respect to metastatic behavior, tumor–microenvironment interactions, and breast cancer subtypes.

Together, our findings indicate that PIEZO1 has a dualistic, subtype-specific role in breast cancer progression, which explains the variable role of PIEZO1 in prognosis across different molecular breast cancer subtypes (4). At the primary site, PIEZO1 supports invasion through RHOA and MMP regulation; however, once the cells are adherent, independent, and in circulation, loss of PIEZO1 is required for survival, stemness, and reduced chemosensitivity in MDA-MB-231 TNBC cells, mediated through β-catenin, pERK1/2, and BCL2 activation. A subtype-specific downregulation of PIEZO1 might inhibit invasion at the primary site, whereas PIEZO1 activation during metastasis could be a potential mechanotherapeutic intervention in breast cancer.

## Supplementary Material

Supplementary Figure 1 Modulation of PIEZO1 activity alters breast cancer cell migration, proliferation, survival under mechanical stress, and sensitivity to pharmacological treatments

## Data Availability

The data generated in this study are available from the corresponding author upon request.
