# Supplementary material for: Downregulation of PIEZO1 Activity Promotes Breast Cancer Cell Survival under Shear Stress by Modulating β-Catenin and BCL2
Source: Cancer Res Commun. 2026 Jul 3;6(7):1557–72. doi: 10.1158/2767-9764.CRC-25-0749 (PMC13329331; doi:10.1158/2767-9764.CRC-25-0749)
Supplement: Supplementary Figure 1 — Modulation of PIEZO1 activity alters breast cancer cell migration, proliferation, survival under mechanical stress, and sensitivity to pharmacological treatments [file crc-25-0749_supplementary_figure_1_suppsf1.pdf]

Supplementary Figure 1

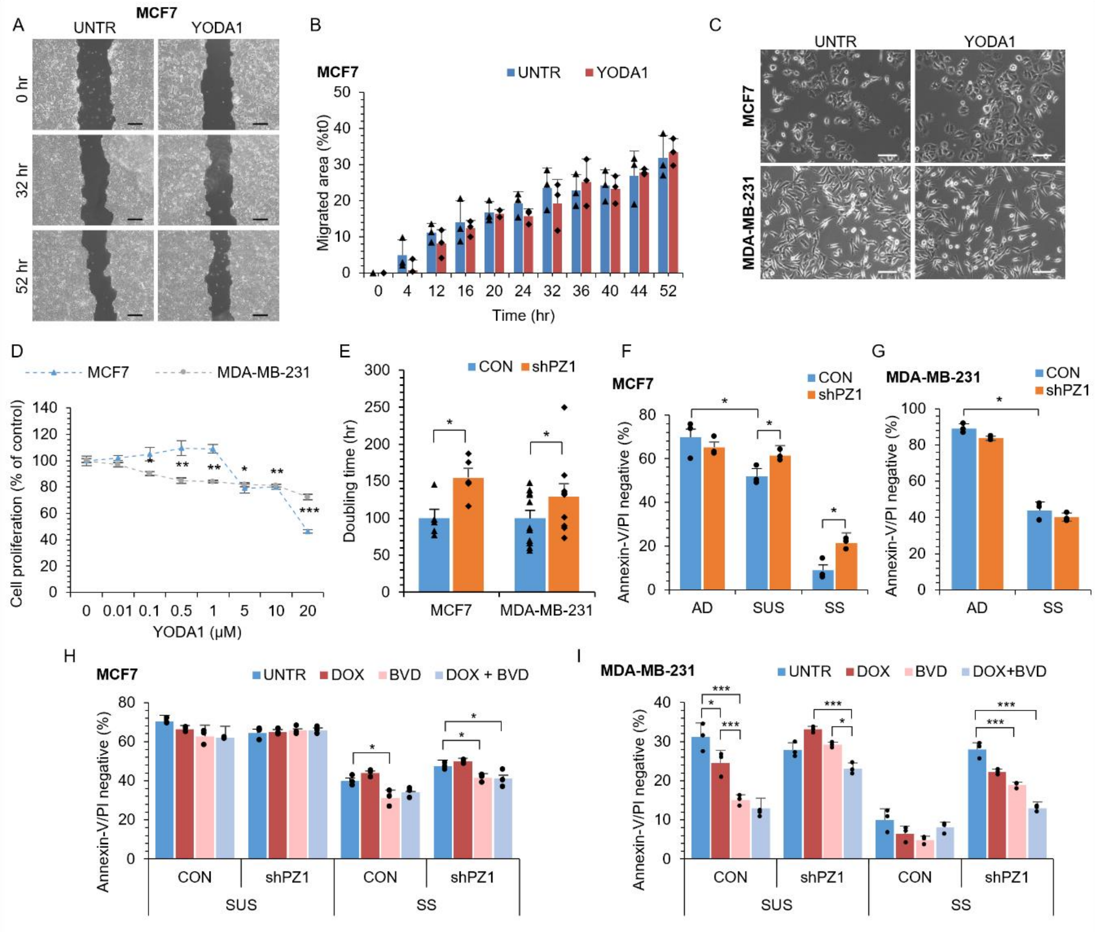

Supp. Fig. 1 Modulation of PIEZO1 activity affects proliferation and survival of breast cancer cells. (A) Representative micrographs of wound healing assay at indicated timepoints showing migration of MCF7 treated with vehicle (UNTR) or 10 µmol/L yoda1 (YODA1). The line in images represents scale bar (200 µm). (B) Migrated area for MCF7 described in A at different time points. Values are mean±S.D. for n=3 with each dot representing reading from a different well. (C) Representative micrographs showing morphology of MCF7 and MDA-MB-231 treated with vehicle (UNTR) or yoda1 (YODA1). The line in images represents the scale bar (100 µm). (D) Cell proliferation of MCF7 and MDA-MB-231 cells treated with different concentration of yoda1 for 72 hours. (E) Doubling time of control (CON) and PIEZO1-silenced (shPZ1) MCF7 and MDA-MB-231. Value are mean+S.E. for n=4-8 passages. (F, G) The cell viability (AnnexinV/PI negative fraction) of control (CON) and PIEZO1-silenced (shPZ1) MCF7 (D) and MDA-MB-231 (E) under static adherent (AD), suspension (SUS), and shear-stress exposed (SS) conditions was assessed by Annexin-V/PI staining. Mean±S.D. for live cell percentages are shown (n=3). (H, I) The Annexin-V/PI negative fraction as percentage of total cells is shown for control (CON) and PIEZO1-silenced (shPZ1) MCF7 (H) and MDA-MB-231 (I) treated with 1 µmol/L doxorubicin (DOX) or 10 µmol/L BVD-523 (BVD) or their combination under static suspension or shear-stress condition. *p* is shown for two-way repeated measures ANOVA with Sidak's multiple comparisons tests in B, one-way ANOVA followed by Dunnett's multiple comparisons test in D, two-way ANOVA with Sidak's test in E-I. *$p<0.05$, **$p<0.005$, ***$p<0.0005$
